# Tim-1-mediated extracellular matrix promotes the development of hepatocellular carcinoma: Tim-1-mediated ECM promotes the development of HCC

**DOI:** 10.3724/abbs.2024191

**Published:** 2024-10-23

**Authors:** Ruheng Hua, Pengfei Yu, Wanting Zheng, Nuwa Wu, Wangjianfei Yu, Qingyu Kong, Jun He, Lei Qin

**Affiliations:** 1 Department of General Surgery the First Affiliated Hospital of Soochow University Suzhou 215006 China; 2 Department of Gastrointestinal Surgery Affiliated Hospital of Nantong University Nantong 226001 China; 3 Affiliated Huishan Hospital of Xinglin College Nantong University Wuxi Huishan District People’s Hospital Wuxi 214100 China; 4 Research Institute of General Surgery Jinling Hospital Nanjing University School of Medicine Nanjing 210095 China

**Keywords:** Tim-1, extracellular matrix, hepatocellular carcinoma, NF-κB, liver fibrosis

## Abstract

Tim-1 (T-cell immunoglobulin and mucin domain 1), also known as Kim-1 (kidney injury molecule 1) or hepatitis A virus cellular receptor 1 (HAVCR1), is a transmembrane protein expressed on various immune and epithelial cells. It plays a role in modulating inflammatory and immune responses. In this study, we find that Tim-1 is overexpressed in hepatocellular carcinoma (HCC) samples and that its expression is significantly correlated with postoperative survival. Bulk RNA sequencing reveals a general upregulation of extracellular matrix-related genes in HCC tissues with Tim-1 overexpression. The results of the cell and *in vivo* experiments reveal that Tim-1 in HCC not only affects biological processes such as the proliferation, migration, and invasion of HCC cells but also broadly promotes extracellular matrix processes by influencing cytokine secretion. Further studies demonstrate that Tim-1 mediates the activation of hepatic stellate cells and upregulates Th1 and Th2 cytokines, thereby promoting HCC progression. Thus, Tim-1 may represent a novel target for future interventions in HCC and liver fibrosis.

## Introduction

Hepatocellular carcinoma (HCC) is the most common primary liver cancer and is characterized by high morbidity and mortality, with multiple predisposing factors contributing to its onset. Research indicates that approximately 90% of patients with HCC have pre-existing liver fibrosis or cirrhosis [1]. As liver fibrosis progress, the extracellular matrix (ECM) is gradually accumulated in liver tissue, leading to mechanical changes and increased stiffness. These ECM alterations contribute to the progression of HCC within a cirrhotic liver environment [ 2, 3].

In an inflammatory environment, studies have shown that Kupffer cells, natural killer cells, and liver sinusoidal endothelial cells secrete significant levels of cytokines such as IL-13, IL-21, and TGF-β. These cytokines stimulate and activate hepatic stellate cells (HSCs), resident fibroblasts, and mesenchymal cells, prompting them to produce increased amounts of type I, III, and IV collagen and laminin [ 4‒ 7]. This process leads to ECM accumulation, supporting the invasion and metastasis of HSCs and liver cancer cells and further promoting the development of HCC [ 8, 9]. Additionally, the ECM serves as the primary structural component of the tumor barrier, significantly hindering the efficacy of chemotherapy drugs against liver cancer cells [ 10, 11].

Tim-1, a member of the human T-cell immunoglobulin and mucin domain (Tim) family, plays a significant role in regulating the tumor microenvironment [ 12‒ 14]. Tim-1 can suppress B-cell activation, antigen presentation, and T-cell activation, negatively impacting antitumor immunity [ 15, 16]. Conversely, blocking Tim-1 reduces IFN-γ production in T cells and effectively decreases the expressions of TNF-α and IL-6, thereby improving the inflammatory tumor microenvironment (TME). Both inflammatory responses and cytokine secretion play crucial roles in the development of HCC and in tumor-related ECM processes [17].

Therefore, investigating and elucidating the impact of Tim-1 on HCC progression, particularly its regulatory influence on the ECM process, is essential for developing novel therapeutic approaches. Our study aims to determine the effect and mechanism of Tim1 in HCC and its clinical value. Our results show that Tim-1 in HCC not only affects biological processes but also broadly promotes extracellular matrix processes by influencing cytokine secretion. Tim-1 mediates the activation of hepatic stellate cells and upregulates Th1 and Th2 cytokines, thereby promoting HCC progression, hinting that Tim1 might be an excellent therapeutic target and prognostic marker for HCC patients.

## Materials and Methods

### Human samples

All human samples were identified and obtained in compliance with local regulations and guidelines. Human liver specimens were collected from the First Affiliated Hospital, Soochow University, with approval from the Institutional Ethics Committee/Institutional Review Board (IEC/IRB) (No. 20130115). Detailed information, including sex, age, diagnosis, and scores, is presented in Table 1.

**Table:** **Table1** Correlation between Tim-1 and clinicopathological characteristics in 156 HCC patients

Variables	Cases	Tim-1 expression	*P*-value
		High (≥medium)	Low (<medium)	
Number	156	78	78	
Age (years)				0.106
<60	88 (56.4%)	39(50.0%)	49(62.8%)	
≥60	68 (43.6%)	39(50.0%)	29(37.2%)	
Gender				0.513
Female	62(39.7%)	29 (37.2%)	33(42.3%)	
Male	94(60.3%)	49 (62.8%)	45(57.7%)	
AFP				0.248
<20	59 (37.8%)	26(33.3%)	33(42.3%)	
≥20	97 (62.2%)	52(66.7%)	45(57.7%)	
Tumor size (cm)				0.006**
<5	77(49.4%)	30(38.5%)	47(60.3%)	
≥5	79 (50.6%)	48(61.5%)	31(39.7 %)	
TNM stage				0.079
I–II	110(70.5%)	50(64.1%)	60(76.9%)	
III	46(29.1%)	28(35.9%)	18(23.1%)	
HbsAg				0.525
Negative	27 (17.3%)	15(19.2%)	12(15.4%)	
Positive	129(82.7%)	63(80.8%)	66(84.6%)	
Embolus				0.037*
Absence	108 (69.2%)	48(61.5%)	60(76.9%)	
Present	48 (30.8%)	30(38.5%)	18(23.1%)	
Hepatic cirrhosis				0.797
No	17 (10.9%)	9(11.5%)	8(10.3%)	
Yes	139(89.1%)	69(88.5%)	70(89.7%)	
Tumor encapsulation				0.010*
None	74(47.4%)	29(37.2%)	45(57.7%)	
Complete	82 (52.6%)	49(62.8%)	33(42.3%)	

^a^Two-sided chi-squared test; HBsAg, hepatitis B surface antigen; AFP, a-fetoprotein. * *P*<0.05, ** *P*<0.01, *** *P*<0.001. Statistically significant values are shown in bold.

### Transcriptome analysis

Samples were sequenced on the platform Novaseq6000 (Illumina, San Diego ,USA) to get image files, which were transformed by the software of the sequencing platform, and the original data in FASTQ format (Raw Data) was generated. Sequencing data contain a number of connectors, low-quality Reads, so fastp (0.22.0) software was used to filter the sequencing data to get high quality sequence (Clean Data) for further analysis. HTSeq (v0.9.1) statistics was used to compare the Read Count values on each gene as the original expression of the gene, and then FPKM was used to standardize the expression. Then difference expression of genes was analyzed by DESeq (v1.38.3) with screened conditions as follows: expression difference multiple |log2FoldChange|>1, significant *P*-value <0.05. At the same time, R language Pheatmap (v1.0.12) software package was used to perform bi-directional clustering analysis of all different genes of samples. We got Heatmap according to the expression level of the same gene in different samples and the expression patterns of different genes in the same sample with Euclidean method to calculate the distance and Complete Linkage method to cluster.

### Animal experiments

All animal experiments were conducted in accordance with humane protocols and were approved by the Animal Ethics Committee of Soochow University (No. 202211A0137). The mice were maintained under optimal conditions, with a temperature range of 20–26°C and humidity levels between 30% and 70%. They were housed in standard cages under a 12/12-h light/dark cycle, with free access to water and food unless otherwise specified. All the mice were purchased from Jiangsu Jicui Yaokang Biotechnology Co., Ltd (Lianyungang, China). To create a subcutaneous tumor model, nude mice were injected with HCC cells that had been transfected with various plasmids. The size of the tumor was assessed at intervals of one week using the formula V=0.5×length×width ^2^. Mice were euthanized after a period of 28 days to measure the weight of tumor tissues. To evaluate liver metastasis, mice were injected with cells that had been transfected with various plasmids through the tail vein. After 6 weeks, the orthotopic liver cancer model was established, then the mice were euthanized and the tumor tissues were collected.

### Cell culture and transfection

The mouse hepatoma cell line Hepa1-6 (CL-0105) was obtained from Wuhan Procell Life Technology Co., Ltd. (Wuhan, China), and the hepatic stellate cell (HSC) line JS1 (BES-3016HC) was obtained from Shanghai BLOESN Biotechnology Co., Ltd. (Shanghai, China). These cells were cultured in DMEM supplemented with 10% fetal bovine serum in an incubator with 95% air and 5% CO _2_ at 37°C. For transfection, cells were prepared using lentiviral vectors, including the Tim-1 overexpression plasmid vector pIRES2-GFP+Puro-Kim1 (PPL00291-2b) and targeted short hairpin RNAs (sh-RNAs) pPLK/GFP+Puro-HAVCR1 sh-RNA (PPL26762) The sequences for shRNAs were 5′-GGAGATTCCTGGATGGTTTAA-3′, both of which were procured from the Public Protein/Plasmid Library (Nanjing, China). Transfection of these plasmids was conducted at room temperature for 48 h using Lipofectamine 3000 (Invitrogen, Carlsbad, USA) according to the manufacturer’s instructions, and 48 h later, the stable transfected cells (Hepa1-6sh-Tim-1 and JS1OE-Tim-1) were selected by 30 days of culture with puro (Genechem, Shanghai, China).

### Western blot analysis

Proteins were extracted from tissue samples and cells using RIPA buffer (Beyotime Biotechnology, Shanghai, China). Then, 20 μg of protein was separated by electrophoresis and transferred to a polyvinylidene fluoride (PVDF) membrane (Millipore, Billerica, USA) at 250 mA for 2 h at 4°C. The membrane was incubated overnight at 4°C with specific antibodies, including antibodies against Tim-1 (sc-518008; 1:1000) and COL1A2 (sc-393573; 1:1000) (Santa Cruz Biotechnology, Santa Cruz, USA), phospho-Akt (Ser473) (1:5000; #4060), Akt (1:1000; #9272), phospho-NF-κB p65 (Ser536) (1:1000; #3033), NF-κB p65 (1:1000; #4764), and GAPDH (1:2000; #5174) (Cell Signaling Technology, Danvers, USA). The membrane was then washed with TBST buffer and incubated with secondary antibody (1:2000; #7074; Cell Signaling Technology) at room temperature for 2 h. The protein bands were visualized and analyzed using a Tanon4160 Chemiluminescence Imager (Beijing, China).

### Cell proliferation assays

For the CCK-8 assay, 100 μL of cell suspension containing 2000 cells per well was added to a 96-well plate. The cells were incubated for 0, 12, 24, 36, or 48 h. Then, 10 μL of CCK-8 solution (Beyotime Biotechnology) was added to each well. After 1 h of incubation, the absorbance was measured at 450 nm using a microplate reader (Thermo Fisher Scientific, Waltham, USA).

### Colony formation assay

After 48 h of transfection, the cells were placed in 6-well plates for incubation. The growth of the culture was halted when cell colonies appeared. Following treatment with formaldehyde and staining with crystal violet for 30 min, the number of colonies was determined using an inverted microscope (Nikon, Tokyo, Japan).

### Wound healing assay

The cells were placed in 6-well dishes and incubated at 37°C for 24 h. Cell transfection was performed following the aforementioned transfection method. At a cell density of 80%–90%, a-200 μL pipette tip was used to scratch a line across the lawn of cells. The cells were washed thoroughly using PBS for 2–3 rounds and then cultured in 1640 medium supplemented with 5% fetal bovine serum (FBS). The closure width/original width of the scratch was observed in real-time using an inverted microscope at 0 h and 48 h. The experiment was conducted three times.

### Flow cytometry

Cell apoptosis was assessed using an Annexin V-FITC Apoptosis Detection kit (Beyotime Biotechnology) according to the manufacturer’s instructions. After staining, the samples were analyzed using a flow cytometer (Beckman Coulter, Pasadena, USA). For tissue cell labeling, the tumor tissue was finely minced and evenly distributed into a six-well plate. Two milliliters of digestive solution (5% DMEM, collagenase I at 1 mg/mL, and deoxyribonuclease I at 200 μg/mL) was added to each well. The plate was incubated in a 37°C cell incubator for 30 min, with shaking every 10 min to ensure proper mixing. The digestion time was strictly followed to preserve immune cell activity. The volume of the digestive solution was adjusted according to the tumor size. After digestion, the plate was placed on ice, and 4 mL of high-glucose DMEM (containing 5% FBS) was added to each well to stop the digestion. The mixture was then filtered through a 70-μm cell strainer, and the remaining tissue was gently ground through the back of a 1-mL syringe. Care was taken not to apply excessive force to avoid introducing fat into the cell suspension. The remaining tissue on the strainer was rinsed with DMEM. The resulting tumor suspension was centrifuged at 850 *g* at 4°C for 5 min. The cell pellet was washed twice with pre-cooled PBS, and 10 μL of monoclonal antibody was dissolved in 200 μL of PBS and then added to the cell pellet. The cells were resuspended and incubated on ice for 30 min. Finally, 300 μL of PBS was added, and the cells were fully resuspended to prepare a single-cell suspension, which was then analyzed by flow cytometry.

### Transwell invasion assay

A total of 600 μL of medium containing 10% FBS was added to the lower chamber, while 100 μL of serum-free medium containing 4×10 ^3^ cells was added to the upper chamber, which was pre-coated with matrix gel. After 12 h of incubation, the invaded cells were fixed with paraformaldehyde, stained with crystal violet, and counted under a microscope (Olympus, Tokyo, Japan).

### Masson staining

First, the sections were routinely dewaxed and rehydrated in water. They were then stained with a prepared Weigert iron hematoxylin solution for 5–10 min, followed by differentiation in an acidic ethanol solution for 5–15 s, and subsequently washed with water. The Mason blue solution was applied again and incubated for 3-5 min, after which the sections were washed with water and rinsed with distilled water for 1 min. This was followed by staining with Ponceau solution for 5–10 min. During these steps, a weak acid working solution was prepared by mixing distilled water with weak acid solution at a 2:1 ratio, and the sections were washed with this solution for 1 min. Next, the sections were cleaned with phosphomolybdic acid solution for 1–2 min, and rinsed with the prepared weak acid working solution for 1 min. The sections were then immersed in aniline blue staining solution for 1–2 min, followed by washing with the weak acid working solution for 1 min. Subsequently, the sections were quickly dehydrated in 95% ethanol for 2–3 s, and then washed with absolute ethanol three times (5–10 s each). Finally, the sections were washed with xylene three times for 1–2 min each, and were sealed with neutral gum.

### IHC staining

IHC staining was performed on formalin-fixed, paraffin-embedded sections. Slides (5–8 μm) were treated with citric acid antigen retrieval solution for 30 min, followed by overnight incubation with anti-Tim-1 (sc-518008; Santa Cruz Biotechnology) at a 1:50 dilution, anti-COL1A2 (sc-393573; Santa Cruz Biotechnology) at a 1:50 dilution, and anti-Ki67 (#9449; Cell Signaling Technology) at a 1:500 dilution. The sections were developed with diaminobenzidine for 10 min, counterstained with hematoxylin, and examined under a microscope (Olympus, Tokyo, Japan). Results were quantified by assessing staining intensity and positivity. Staining intensity was scored as follows: H-Score [H-Score=∑(pi×i)=(percentage of weak intensity×1)+(percentage of moderate intensity×2)+(percentage of strong intensity×3], where pi represents the ratio of positive signal pixel area to cell number; I represents coloring intensity). The H-Score ranges from 0 to 300, and the larger the data are, the stronger the overall positive intensity.

### Statistical analysis

Data are expressed as the mean±standard deviation (SD). Statistical analyses were conducted using GraphPad Prism 8.0 (GraphPad Software, San Diego, USA). Differences between groups were assessed using Student’s *t* test or ANOVA. *P*<0.05 was considered statistically significant.

## Results

### High expression of Tim-1 in HCC indicates poor survival

**Figure 1:**
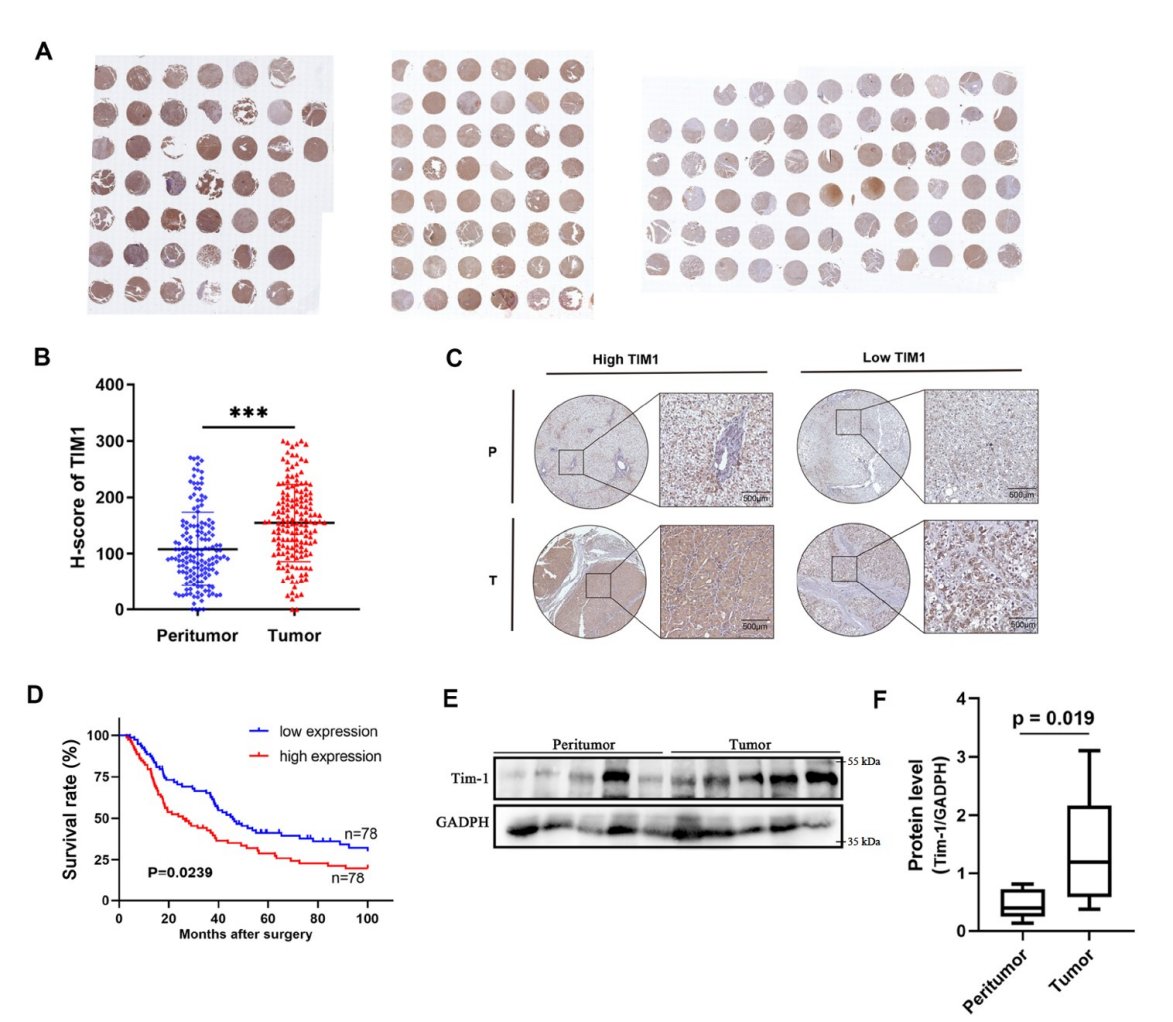
Figure 1 High expression of Tim-1 in HCC is correlated with poor survival (A) Tumour tissue samples from 156 liver cancer patients were subjected to immunohistochemistry (IHC) with a Tim-1 antibody according to the manufacturer’s instructions. (B) H score analysis based on (A). (C) IHC staining data distinguishing between low and high Tim-1 expression in tumor samples. (D) Five-year survival rates were assessed in patients following surgery. (E) Tumor tissues and adjacent tissues were collected during surgery and subjected to western blot analysis with a Tim-1 antibody. (F) Tim-1 expression in (E) was quantified by calculating relative grayscale values via ImageJ. ***P<0.001.

We collected 156 cases of hepatocellular carcinoma (HCC), and Table 1 provides the relevant patient information. IHC staining of Tim-1 was performed on pathological tissue sections from the case samples ( Figure 1A). H-score analysis was conducted to evaluate Tim-1 levels in both tumor and adjacent tissues from 156 patients. The results revealed a significant increase in Tim-1 expression within tumors compared with adjacent tissues ( Figure 1B). Patients were then categorized into high- and low-Tim-1 expression groups on the basis of the IHC results ( Figure 1C), and their postoperative survival was monitored. Patients with high Tim-1 expression had a significantly lower 5-year survival rate than those with low Tim-1 expression ( Figure 1D). Randomly selected tumor and adjacent tissue samples were subjected to western blot analysis, revealing a significant increase in Tim-1 levels in tumor tissues compared with adjacent tissues ( Figure 1E,F). These data suggested that Tim-1 plays a critical role in HCC and impacts postoperative survival.

### Aberrant expression of Tim-1 in HCC is closely associated with the tumor ECM

**Figure 2:**
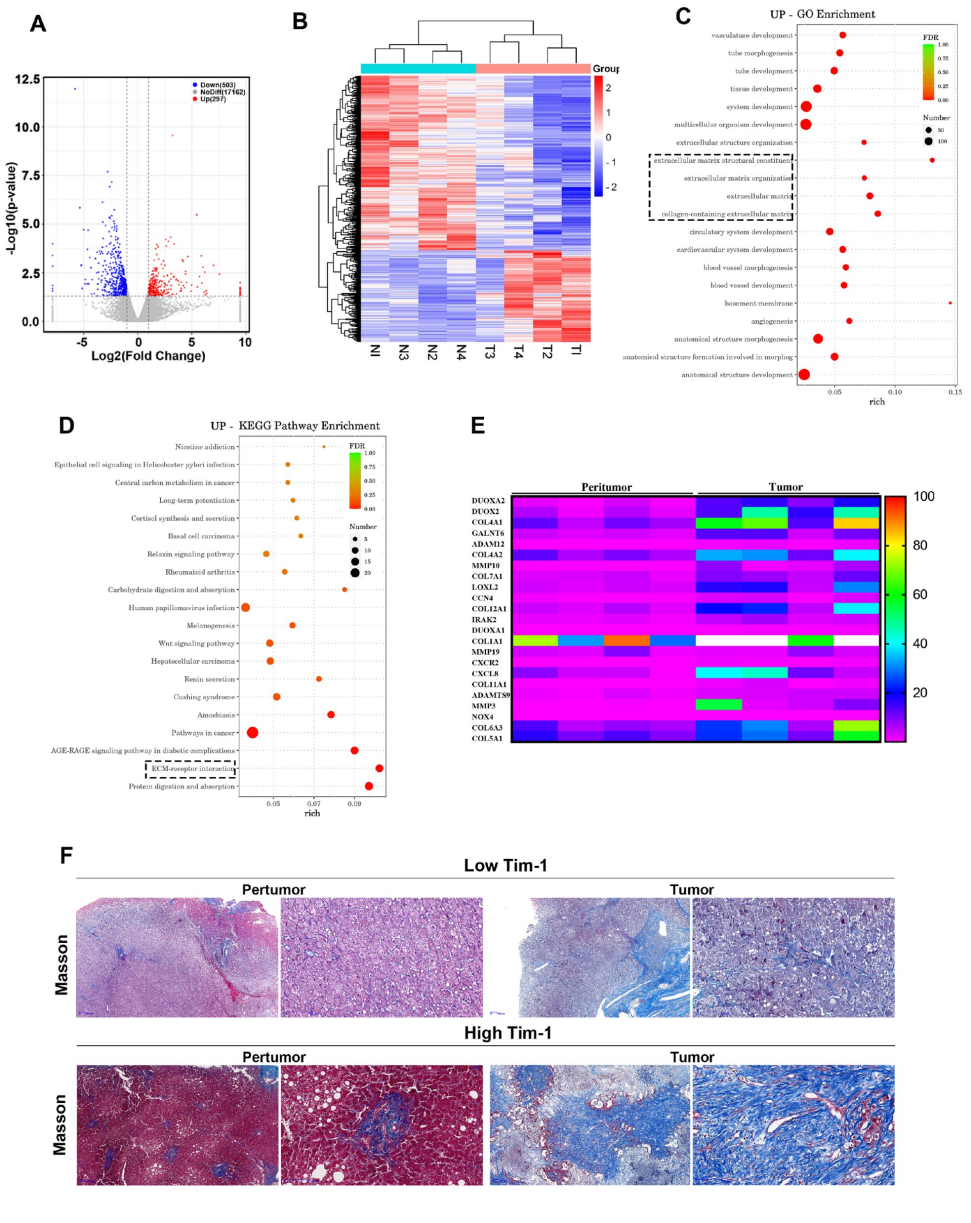
Figure 2 Aberrant Tim-1 expression in HCC is closely linked to the tumor ECM (A) Analysis of bulk RNA sequencing data from tumor and adjacent tissues from patients with high Tim-1 expression (n= 4 each). (B) Heatmap showing upregulated and downregulated genes. (C) GO enrichment analysis of up-regulated transcriptional or splicing gene categories in peritumor and HCC tumor tissues. (D) KEGG pathway enrichment analysis of upregulated transcriptional or splicing gene categories in peritumor and HCC tumor tissues. (E) Heatmap displaying upregulated ECM-related genes. (F) Tumor tissues from patients with low and high Tim-1 expression were subjected to Masson staining to assess the severity of the ECM associated with intrahepatic tumors.

To further investigate the role of Tim-1 in HCC, bulk RNA sequencing was performed on both adjacent non-cancerous and cancerous tissues from patients with high Tim-1 expression. The analysis revealed that 297 genes were upregulated and 503 genes were downregulated in these cancerous tissues ( Figure 2A,B). GO enrichment analysis of the upregulated genes revealed a significant increase in ECM-related genes ( Figure 2C). KEGG signaling pathway enrichment analysis also highlighted an interesting observation regarding ECM-receptor interactions ( Figure 2D). Further analysis of these ECM-related genes revealed widespread upregulation of collagen family genes (COLs) in patients with high Tim-1 expression ( Figure 2E). Additionally, Masson staining of liver tissues from some patients revealed more severe collagen fibre deposition in specimens with high Tim-1 expression ( Figure 2F), indicating a potential link between high Tim-1 expression and intrahepatic ECM deposition in HCC patients.

### Blocking Tim-1 inhibits HCC-driven ECM

**Figure 3:**
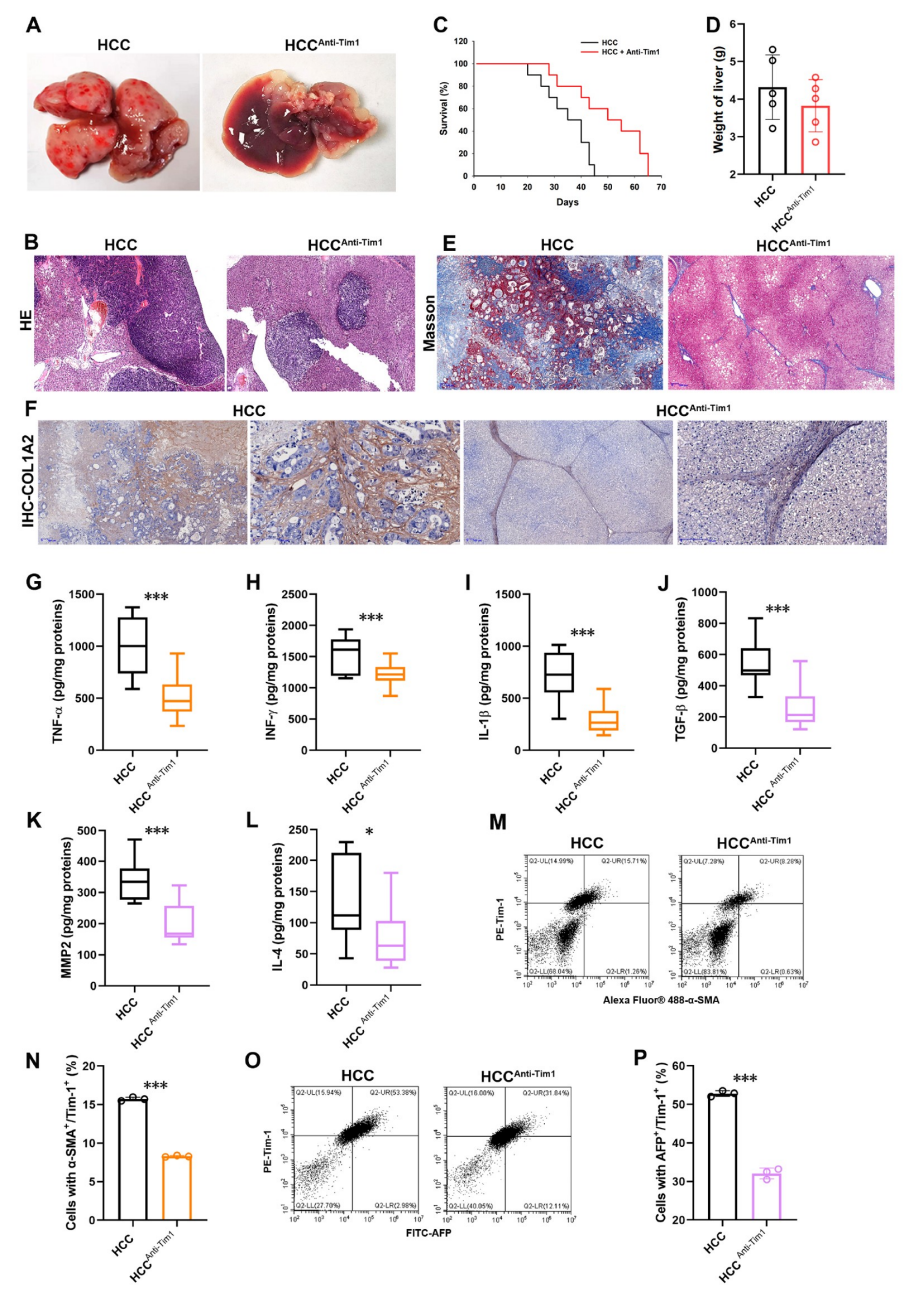
Figure 3 Blocking Tim-1 inhibits HCC-driven ECM A total of 107 Hepa1-6 cells were transplanted into mice via intravenous injection to establish an HCC model. Five days after transplantation, Tim-1 monoclonal antibody (5 μg per 20 g of mouse) was injected into the mice every two days for a total of five injections (n =10 each). (A) Liver morphology of mice transplanted with Hepa1-6 cells after 25 days and liver morphology of HCC mice after treatment with a Tim-1 monoclonal antibody ( n=5 each). (B) HE staining of liver tissues was performed to observe the infiltration of liver cancer cells. (C) Survival curves were used to estimate the survival period of the mice (n=5 each). (D) Liver weight after 25 days (n=5 each). (E) Masson staining of liver tissues after 25 days (n =5 each). (F) IHC staining with an anti-COL1A2 antibody was used to assess collagen generation in liver tissues. (G‒L) The contents of liver tissues were extracted to detect intracellular Th1-type cytokines, such as TNF-α (G), INF-γ (H), and IL-1β (I), and Th2-type cytokines, such as the cytokines TGF-β (J), MMP2 (K), and IL-4 (L), via ELISA kits. (M‒P) Intrahepatic cells were extracted and labelled with a PE-Tim-1 antibody and an Alexa Fluor-488-α-SMA antibody (M,N) or with a PE-Tim-1 antibody and a FITC-AFP antibody (O,P). These labelled cells were analyzed by flow cytometry to assess changes in HSCs and hepatoma cells. *P<0.05, ***P<0.001.

To explore the impact of Tim-1 on the ECM in HCC, we established an orthotopic liver cancer model by injecting Hepa1-6 cells into female mice via the tail vein. Subsequently, an anti-Tim-1 monoclonal antibody (RMT1-10) was administered intraperitoneally to inhibit the effects of Tim-1 [18]. Liver tissue images confirmed successful infiltration of Hepa1-6 cells into the mouse liver, leading to cancer development. However, blocking Tim-1 significantly delayed this progression ( Figure 3A). Hematoxylin and eosin (HE) staining of the relevant tissues revealed substantial cancer cell infiltration in the liver tissue, which was notably reduced following Tim-1 inhibition ( Figure 3B). Blocking Tim-1 also prolonged the survival of the mice ( Figure 3C), although there was no significant change in liver weight ( Figure 3D). Masson staining of liver tissue revealed a high presence of collagen fibers in areas infiltrated by tumor cells, with a significant reduction in collagen fiber after Tim-1 blockade, indicating that Tim-1 expression in HCC contributes to ECM production in the liver ( Figure 3E). IHC staining for type I collagen alpha 2 chain (COL1A2) revealed that Hepa1-6-infiltrating HCC cells expressed abundant COL1A2, but blocking Tim-1 significantly inhibited collagen invasion into the liver parenchyma ( Figure 3F). Additionally, liver tissue extracts were analyzed for intracellular Th1-type cytokines (TNF-α, INF-γ, and IL-1β) and Th2-type cytokines (TGF-β, MMP2, and IL4) using ELISA kits ( Figure 3G‒L). The results showed a significant downregulation of these cytokines following Tim-1 inhibition, suggesting a strong correlation between elevated Tim-1 expression and cytokine secretion in liver cancer, potentially influencing ECM augmentation. Considering the crucial role of hepatic stellate cells (HSCs) in the liver ECM process, we used α-SMA and Tim-1 monoclonal antibodies to identify HSCs in liver tissue and alpha-fetoprotein (AFP) and Tim-1 to identify liver cancer cells [ 19, 20]. Figure 3M‒P revealed a notable reduction in the number of α-SMA and Tim-1 double-positive cells in liver cancer tissues by approximately 1-fold, whereas the number of AFP and Tim-1 double-positive cells was reduced by approximately 40%. These findings suggested a strong correlation between elevated Tim-1 expression in liver cancer, HSC activation, increased ECM, and the potential therapeutic impact of Tim-1 blockade on HCC progression.

### Tim-1 plays a crucial role in mediating the proliferation and migration of HCC cells

**Figure 4:**
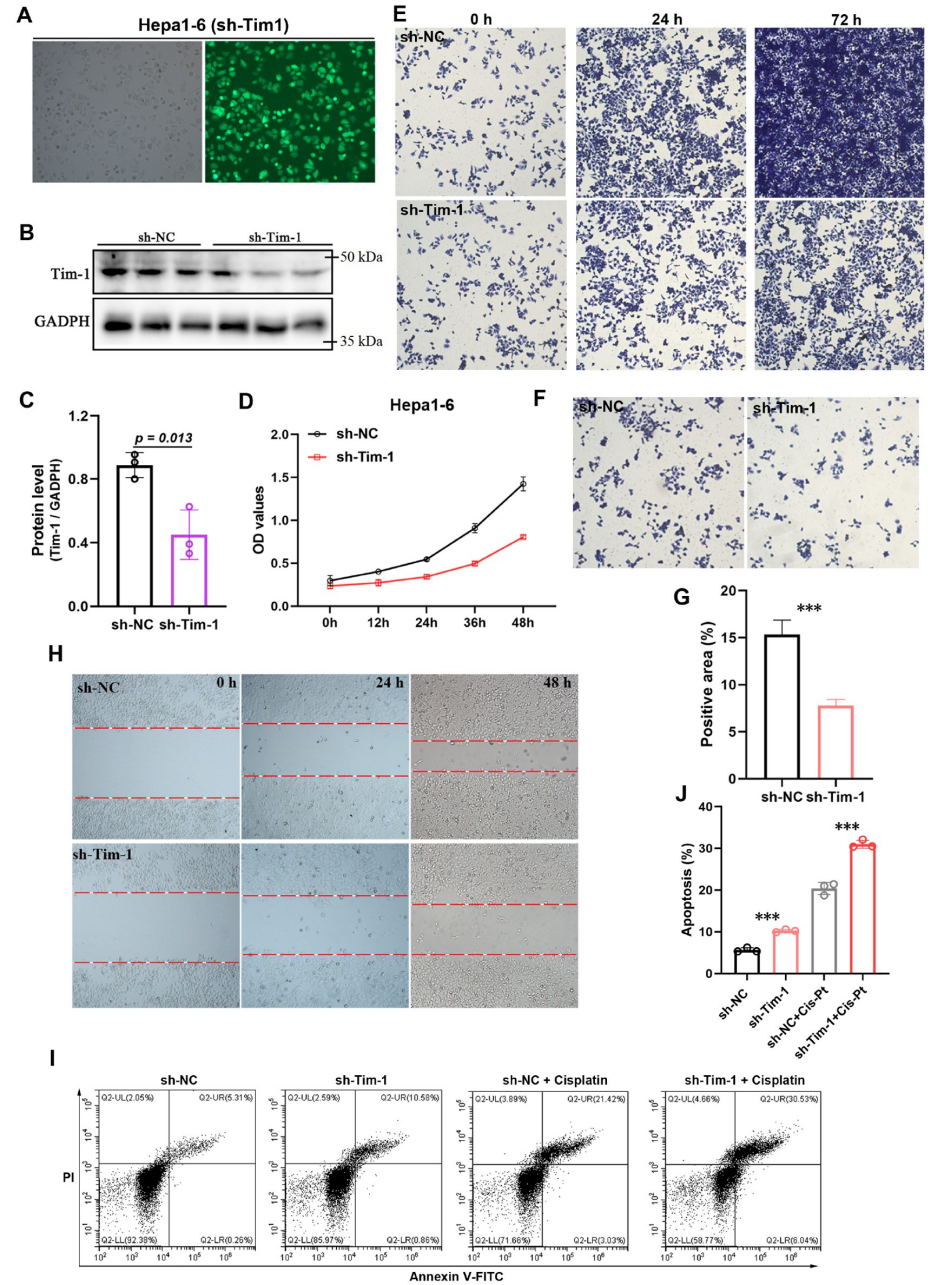
Figure 4 Tim-1 plays a crucial role in mediating the proliferation and migration of HCC cells A shRNA plasmid targeting Tim-1 expression was constructed and introduced into Hepa1-6 cells using lentivirus. (A) Fluorescence microscopy image showing stable knockdown of Tim-1 in Hepa1-6 cells. (B) Western blot analysis of Tim-1 protein confirming that the Tim-1 gene was knocked down. (C) The level of Tim-1 protein was quantified by calculating the relative grayscale values via the image analysis software ImageJ. (D,E) CCK8 signal abundance detection (D) and crystal violet staining (E) were performed to assess cell proliferation prior to and subsequent to Tim-1 protein downregulation. (F) Transwell experiments were performed to assess the impact of pre- and post-Tim-1 knockdown on cell invasion. (G) Quantification of the stained cell area in (F) using ImageJ. (H) An experimental design was used to assess the influence of Tim-1 gene knockdown, both before and after intervention, on the migratory capacity of the cells. (I) Flow cytometry was used to assess the effects of Tim-1 knockdown on cell apoptosis and the toxicity of cisplatin. (J) Quantification of the percentage of apoptotic cells in (I). ***P<0.001 (n=3 each).

As an immune co-stimulatory molecule, Tim-1 not only enhances T-cell activation and proliferation and regulates cytokine secretion but is also overexpressed in various cancers, facilitating tumor progression. Research indicates that Tim-1 overexpression in tumors promotes cell migration and invasion via the MEK/ERK and PI3K/AKT signaling pathways. To elucidate the role of Tim-1 in HCC, a shRNA plasmid targeting Tim-1 mRNA was constructed and introduced into Hepa1-6 cells via a lentivirus. After screening, Hepa1-6 cells with *Tim-1* knockdown (Hepa1-6 ^sh-Tim-1^) were obtained ( Figure 4A). Western blot analysis confirmed a substantial decrease in Tim-1 protein level in Hepa1-6 ^sh-Tim-1^ cells ( Figure 4B,C). The results of the CCK-8 assay indicated that *Tim-1* knockdown suppressed Hepa1-6 cell proliferation ( Figure 4D). Crystal violet staining further confirmed the reduced proliferation of Hepa1-6 ^sh-Tim-1^ cells ( Figure 4E). Additionally, both transwell ( Figure 4F,G) and scratch ( Figure 4H) assays demonstrated that *Tim-1* knockdown significantly inhibited the invasion and migration of Hepa1-6 cells. Furthermore, while *Tim-1* knockdown slightly increased cell apoptosis, it significantly increased the degree of apoptosis induced by cisplatin, a standard chemotherapy drug for HCC ( Figure 4J,I). These results suggested that Tim-1 plays a broad role in HCC by mediating cell proliferation, migration, invasion, and apoptosis.

### *Tim-1* knockdown inhibits the progression of orthotopic HCC

**Figure 5:**
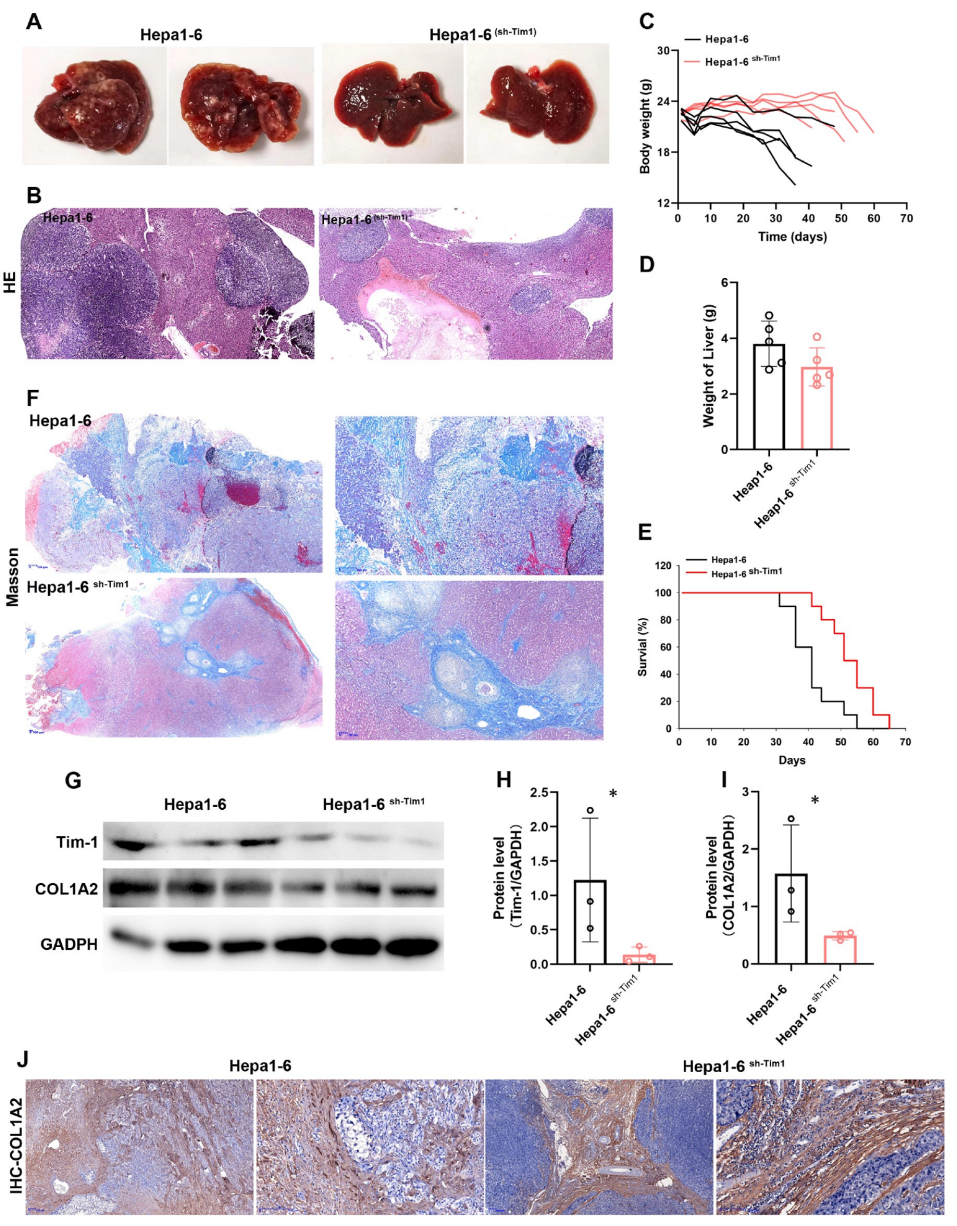
Figure 5 Knockdown of *Tim-1* inhibits the progression of orthotopic HCC To establish a HCC mouse model, 107 Hepa1-6 cells and 107 Hepa1-6sh-Tim-1 cells were intravenously injected into mice (n=10 each). (A) Following a period of 25 days, the liver morphologies of Hepa1-6 cells transplanted into mice were evaluated (n=5 each). (B) HE staining of liver tissue sections. (C) Changes in body weight (n=5 each). (D) Liver weight after 25 days (n=5 each). (E) Survival period of the mice (n=5 each). (F) Masson staining of liver tissue sections after 25 days (n=5 each). (G) Liver tissues were extracted and subjected to western blot analysis with COL1A2 and Tim-1 antibodies. (H,I) Quantification of Tim-1 and COL1A2 expressions in (G) by calculating the relative grayscale values using ImageJ software. (J) IHC staining of COL1A2 in liver tissue sections was used to estimate the generation of collagen proteins in liver tissues.

To further confirm the influence of Tim-1 on HCC, Hepa1-6 and Hepa1-6 ^sh-Tim-1^ cells were injected into mice via the tail vein to establish an HCC model. Compared with those formed by Hepa1-6 cells, the liver tissues formed by Hepa1-6 ^sh-Tim-1^ infiltration presented significantly milder symptoms ( Figure 5A). HE staining of liver tissues revealed a greater density of cancer cells infiltrated by Hepa1-6 cells, whereas there was a notable reduction in infiltration by Hepa1-6 ^sh-Tim-1^ cells ( Figure 5B). Additionally, mice infiltrated with Hepa1-6 ^sh-Tim-1^ exhibited less weight change and a decrease in liver weight than mice infiltrated with Hepa1-6 ( Figure 5C,D). In terms of survival, *Tim-1* knockdown significantly extended the lifespan of the mice ( Figure 5E), similar to the effects of Tim-1 antibody blockade. Masson staining of liver tissues further demonstrated that *Tim-1* knockdown effectively suppressed the generation of intrahepatic collagen fibers, indicating its impact on the ECM during liver cancer progression ( Figure 5F). Western blot analysis revealed that Tim-1 inhibition reduced COL1A2 protein level in the liver ( Figure 5G‒I), which is consistent with the findings of COL1A2-stained IHC ( Figure 5J). *Tim-1* knockdown significantly reduced collagen protein expression in the liver parenchyma, although it remained abundant in the liver sinusoids. These findings suggested that *Tim-1* is a pivotal gene influencing HCC progression, particularly by regulating the ECM during cancer progression.

### Tim-1 increases the secretion of cytokines in hepatic stellate cells via NF-κB-related signaling pathways

**Figure 6:**
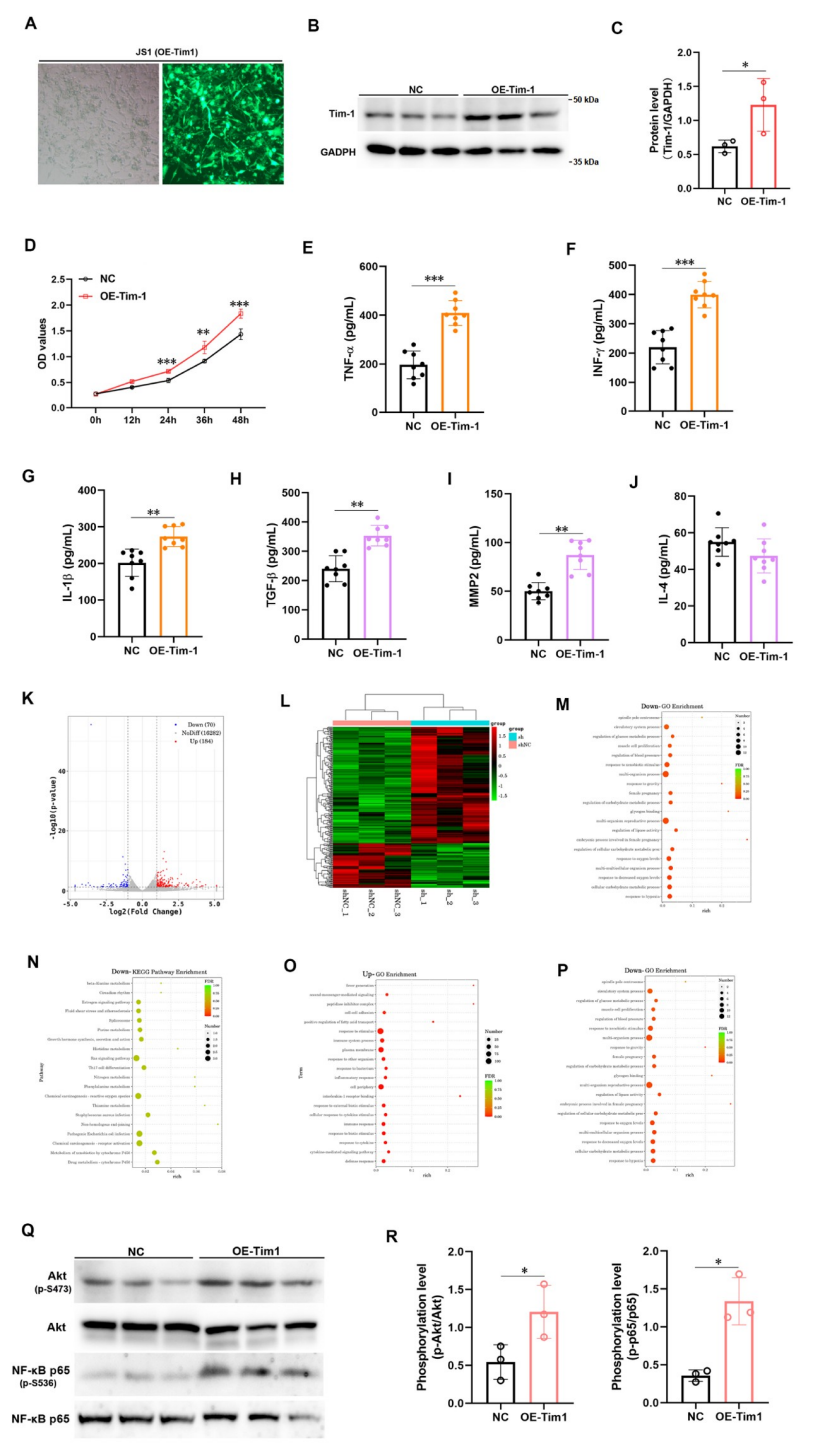
Figure 6 Tim-1 enhances cytokine secretion in hepatic stellate cells via NF-κB-related signaling pathways A plasmid construct designed to overexpress Tim-1 was generated and transduced into the JS1 cell line via lentivirus. (A) Fluorescence microscopy examination of Tim-1 protein expression in the JS1 cell line following stable overexpression. (B) Western blot analysis confirmed Tim-1 protein overexpression. (C) Quantification of Tim-1 protein levels in (B) by calculating the relative grayscale values using ImageJ. (D) CCK8 assay was used to evaluate cell proliferation before and after Tim-1 overexpression. These Tim-1-overexpressing cells were analyzed for the levels of intracellular Th1-type TNF-α (E), INF-γ (F), and IL-1β (G) and Th2-type TGF-β (H), MMP2 (I), and IL-4 (J) using corresponding ELISA kits. A comprehensive analysis of the bulk RNA-seq data obtained from JS1 cells displaying high Tim-1 expression was performed (n=3 each). (K,L) Heatmaps showing upregulated and downregulated genes. (M,O) GO enrichment analysis of upregulated and downregulated genes. (N,P) KEGG pathway enrichment analysis of upregulated and downregulated genes. (Q) JS1 cells with high Tim-1 expression were subjected to western blot analysis with antibodies against phosphorylated Akt, phosphorylated NF-κB p65, Akt, and NF-κB p65. (R) Quantification of the relative levels of phosphorylated Akt and phosphorylated NF-κB p65 in (Q) by calculating the relative grayscale values via ImageJ. **P<0.01; ***P<0.001.

Tim-1 blockade was observed to decrease the proportion of α-SMA and Tim-1 double-positive cells in a mouse liver cancer model, suggesting a regulatory role of Tim-1 in HSC activation. To investigate the impact of Tim-1 on HSCs, we constructed an overexpression plasmid for Tim-1 (OE-Tim-1) and introduced it into JS1 cells using lentivirus-mediated stable transfection ( Figure 6A). The western blot analysis results revealed significant upregulation of intracellular Tim-1 ( Figure 6B,C). The results of the CCK-8 assay indicated that Tim-1 overexpression increased JS1 cell proliferation ( Figure 6D). Additionally, ELISA kits were used to assess the secretion of Th1-type cytokines (TNF-α, INF-γ, and IL-1β) and Th2-type cytokines (TGF-β, MMP2, and IL4) in the cell supernatant, revealing significant upregulation of all six cytokines ( Figure 6E‒J). Bulk RNA sequencing revealed an increase in 184 gene transcripts and a decrease in 70 gene transcripts ( Figure 6K,L). Subsequent GO enrichment analysis and KEGG signaling pathway analysis revealed notable downregulation of genes associated with oxygen level, as well as general downregulation of the Ras signaling pathway, the ROS signaling pathway, and Th17 differentiation signals ( Figure 6M,N). Conversely, the upregulated genes were enriched in groups related to cell adhesion, the inflammatory response, the immune response, and the cytokine response, with a significant impact on the NF-κB signaling pathway ( Figure 6O,P). Previous studies have shown that Tim-1 plays a role in activating the Akt-NF-κB signaling pathway [ 16, 21 ]. We analyzed the activation of Akt/NF-κB signaling in cells overexpressing Tim-1 and observed a notable increase in phosphorylated Akt and phosphorylated NF-κB p65 levels after Tim-1 overexpression. These data indicated that Tim-1 enhances NF-κB signaling activation in JS1 cells, thereby stimulating the secretion of various cytokines ( Figure 6 Q,R).

### High expression of Tim-1 in HSCs drives the progression of HCC by enhancing the ECM

**Figure 7:**
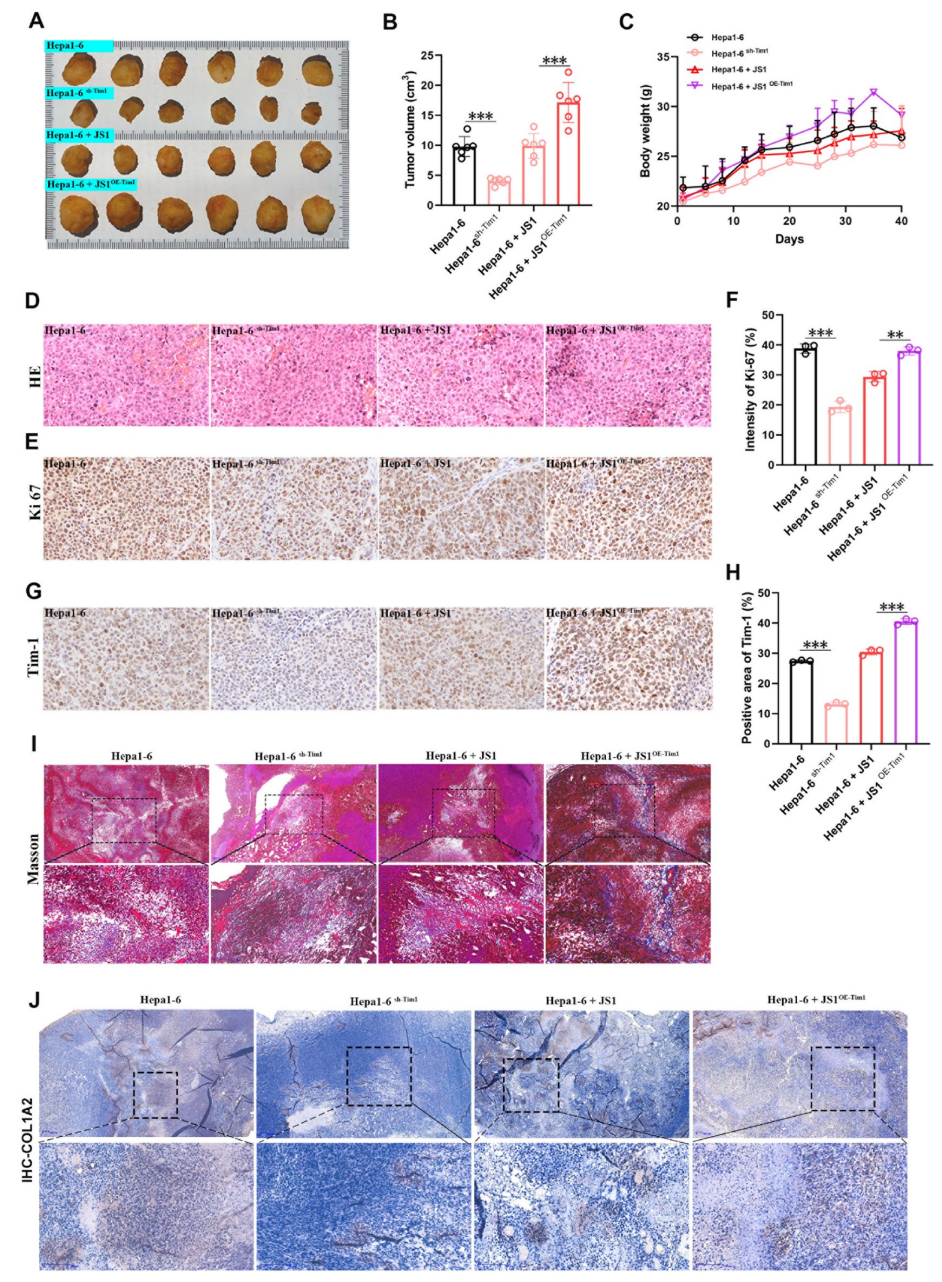
Figure 7 High expression of Tim-1 in HSCs drives HCC progression by enhancing the ECM A total of 107 Hepa1-6 cells, 107 Hepa1-6sh-Tim-1 cells, 107 Hepa1-6+105 JS1 cells, and 107 Hepa1-6+105 JS1 OE-Tim-1 cells were injected into the subcutaneous tissues of BALB/c nude mice to establish subcutaneous tumors (n=10 each). (A) Sarcoma tissues were harvested during a 25-day proliferation period postcell engraftment, and the histopathological characteristics and sizes of the sarcoma tumors were comprehensively evaluated (n=5 each). (B) Sarcoma tissue volume (n=5 each). (C) Changes in body weight (n=5 each). (D) HE staining of liver tissue sections. (E) IHC staining with Ki67 antibody was used to assess the proliferative ability of sarcoma tissues. (F) Quantification of Ki67-positive cells via ImageJ. (G) IHC staining with Tim-1 antibody was used to assess Tim-1 levels in these sarcoma tissues. (H) Quantification of Tim-1 levels using ImageJ. (I) Masson staining of these sections of sarcoma tissues. (J) IHC staining with an anti-COL1A2 antibody was used to assess collagen protein levels in these sarcoma tissues. ** P<0.01, ***P<0.001.

To further confirm whether Tim-1 promotes HCC development by mediating cancer cell proliferation and regulating the ECM, we conducted an experiment in which 10 ^7^ Hepa1-6 cells, 10 ^7^ Hepa1-6 ^sh-Tim-1^ cells, 10 ^7^ Hepa1-6+10 ^5^ JS1 cells, and 10 ^7^ Hepa1-6+10 ^5^ JS1 ^OE-Tim-1^ cells were injected into the subcutaneous tissue of nude mice to establish a subcutaneous tumor model. Twenty-five days after transplantation, the mice were sacrificed, and the sarcoma tissues were collected. We observed that tumor growth was notably slower following the transplantation of Hepa1-6 ^sh-Tim-1^ cells than following the transplantation of Hepa1-6 cells, highlighting the significant role of Tim-1 overexpression in accelerating tumor progression ( Figure 7A,B). Interestingly, co-transplantation of Hepa1-6/JS1 cells did not affect tumor size, whereas co-transplantation of Hepa1-6/JS1 ^OE-Tim-1^ cells notably enhanced tumor progression ( Figure 7A,B). Furthermore, changes in body weight indicated that co-transplantation of Hepa1-6/JS1 ^OE-Tim-1^ led to an increase in body weight, whereas the slowest weight gain was observed after Hepa1-6 ^sh-Tim-1^ transplantation ( Figure 7C).

Some tumor tissues were sectioned and stained with HE staining, revealing a large concentration of tumor cells in the sarcoma tissue ( Figure 7D). IHC staining for Ki67 and Tim-1 revealed that downregulation of Tim-1 in transplanted tumors corresponded with reduced Ki67 expression, whereas Tim-1 overexpression in JS1 cells promoted Ki67 expression in sarcoma tissue ( Figure 7E‒H). Additionally, Masson staining of the relevant tissues revealed that the combined transplantation of Hepa1-6/JS1 ^OE-Tim-1^ cells significantly increased collagen fiber production ( Figure 7I). IHC staining for COL1A2 revealed similar results ( Figure 7J), further indicating minimal cancer cell infiltration and collagen fiber formation in the liver following Hepa1-6 ^sh-Tim-1^ transplantation. In contrast, the combined transplantation of Hepa1-6/JS1 ^OE-Tim-1^ resulted in the significant presence of both cancer cells and collagen fibers. These findings underscore the critical role of Tim-1 in HCC, not only in promoting cancer cell proliferation but also in regulating the ECM and influencing HCC progression.

## Discussion

The development of HCC is closely linked to liver fibrosis and cirrhosis, with approximately 70% to 90% of HCC patients exhibiting underlying liver fibrosis [22]. Extensive evidence suggests that HSCs play a significant role in the initiation and progression of HCC, and it is widely believed that fibroblasts within HCC originate from HSCs [ 23‒ 25]. Activated HSCs release various proteins, including vimentin, ECM proteins, and α-SMA, leading to the secretion of inflammatory factors and cytokines that worsen the inflammatory microenvironment and exacerbate ECM formation. Cytokines such as TGF-β1 stimulate the expression of α-SMA in activated HSCs and upregulate IL-1 and vascular endothelial growth factor (VEGF), thereby enhancing HCC invasion and metastasis [ 26, 27]. Additionally, increased ECM in the liver not only facilitates cancer cell proliferation and metastasis but also acts as a physical barrier that hinders drug diffusion within the tumor, reducing the efficacy of tumor cell eradication [ 28, 29].

The inflammatory TME plays a critical role in promoting the tumor-associated ECM. The ECM is a non-cellular, three-dimensional macromolecular network in which collagen, fibronectin, laminin, and other matrix glycoproteins contribute to the structural framework of cancer fibrosis and solid tumors [30]. The biosynthesis of collagen is intricately regulated by fibroblasts, cancer cells, and stromal cells such as macrophages [31]. Numerous studies have shown that the tumor-associated ECM promotes tumor cell growth, invasion, metastasis, angiogenesis, and resistance to cell death and drug penetration [32]. However, in certain tumors, collagen fibers within the interstitial matrix become thicker, more organized, and denser due to increased collagen deposition. As cancer progresses, stromal collagen fibers become more orderly, particularly at tumor borders, facilitating cancer cell invasion.

Although Tim-1 plays a crucial role in regulating immunity, hepatic virus invasion, and kidney damage, few studies have focused on its role in HCC development [ 13, 33, 34]. In this study, we demonstrated that Tim-1 mediates tumor cell proliferation and migration in HCC, regulates HSC activation, and influences ECM formation. As a receptor for hepatitis A virus, Tim-1 functions as a costimulatory molecule that interacts with the Tim-4 ligand to activate helper T 2 (Th2) cells and facilitate tumor progression [35]. Research indicates that Tim-1 is significantly expressed in human non-small cell lung cancer tissues and that knocking down *Tim-1* reduces cell viability, migration, and invasion by suppressing the PTEN/Akt pathway. Additionally, Akt-NF-κB signaling plays a role in HSC activation [36]. These findings suggested that the upregulation of Tim-1 in liver cancer may enhance ECM processing through the Akt-NF-κB pathway. Treatment with anti-Tim-1 agents has also been shown to inhibit NF-κB activation and apoptosis, while Tim-1 inhibition plays a crucial role in preventing cisplatin-induced acute kidney injury, as *Tim-1* is also a key gene in regulating renal inflammation and fibrosis [ 21, 37‒ 39] .

Targeting the overexpression of Tim-1 may offer a promising therapeutic approach in HCC. Inhibiting Tim-1 could reduce HCC proliferation, enhance the apoptotic effects of chemotherapy drugs, and hinder the ECM process, thereby slowing tumor progression and metastasis. Additionally, reducing the ECM may improve chemotherapy efficacy and combat drug resistance by lowering the biological barriers surrounding tumors. Unfortunately, there are currently no reported inhibitors specifically targeting Tim-1.
